# Contrast Improvement in Sub- and Ultraharmonic Ultrasound Contrast Imaging by Combining Several Hammerstein Models

**DOI:** 10.1155/2013/270523

**Published:** 2013-11-07

**Authors:** Fatima Sbeity, Sébastien Ménigot, Jamal Charara, Jean-Marc Girault

**Affiliations:** ^1^Université François Rabelais de Tours, UMR-S930, 37032 Tours, France; ^2^IUT Ville d'Avray, Université Paris Ouest Nanterre La Défense, 92410 Ville d'Avray, France; ^3^Département de Physique et d'Électronique, Faculté des Sciences I, Université Libanaise, Hadath, Lebanon

## Abstract

Sub- and ultraharmonic (SUH) ultrasound contrast imaging is an alternative modality to the second harmonic imaging, since, in specific conditions it could produce high quality echographic images. This modality enables the contrast enhancement of echographic images by using SUH present in the contrast agent response but absent from the nonperfused tissue. For a better access to the components generated by the ultrasound contrast agents, nonlinear techniques based on Hammerstein model are preferred. As the major limitation of Hammerstein model is its capacity of modeling harmonic components only, in this work we propose two methods allowing to model SUH. These new methods use several Hammerstein models to identify contrast agent signals having SUH components and to separate these components from harmonic components. The application of the proposed methods for modeling simulated contrast agent signals shows their efficiency in modeling these signals and in separating SUH components. The achieved gain with respect to the standard Hammerstein model was 26.8 dB and 22.8 dB for the two proposed methods, respectively.

## 1. Introduction

**Figure 1:**
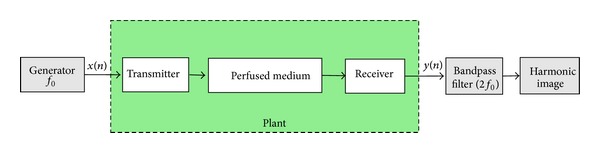
Block diagram of second harmonic imaging.

Introduction of contrast agents in ultrasound medical imaging has strongly improved the image contrast leading to a better medical diagnosis [1–3]. By adapting the transmitting ultrasound sequences composed of short wave trains to longer sinusoidal wave trains, it has been possible to enhance the harmonics detection witnessing of the presence of nonlinear explored media [3–5]. The most prominent example in echographic imaging is the second harmonic imaging (SHI) [3, 6] which consists to send a sinusoidal wave train of frequency *f*_0_ and to receive the backscattered signal at twice the transmitted frequency, that is, 2*f*_0_ (see Figure 1).

**Figure 2:**
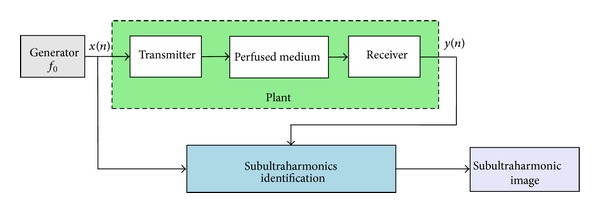
Block diagram of subultraharmonic imaging.

 Although the second harmonic imaging possesses undoubted advantages compared to standard echographic imaging, contrast harmonic imaging, however, has image contrast limitations related to the presence of harmonic components of nonlinear nonperfused tissues [7]. This contrast reduction can be overcome by proposing no more contrast harmonic imaging but rather contrast subultraharmonic (SUH) imaging [8, 9]. Under certain conditions of incident frequency and pressure levels, this solution has been envisaged as a serious alternative [10, 11] since it has been shown that only contrast agent is capable of supplying SUH components sufficient to construct perfused tissue images with a strong contrast. Contrast SUH imaging consists to send a sinusoidal wave train of frequency *f*_0_ and to extract from the backscattered signal only SUH frequencies at *f*_0_/2, (3/2)*f*_0_, (5/2)*f*_0_,… (see Figure 2).

 To extract such SUH components from the whole spectrum, a certain number of approaches called “black box methods” has been proposed such as those based on the multiple input and single output (MISO) Volterra filtering [12–14]. These recent methods are capable of accurately modeling the signal backscattered by the contrast agent with adequate values of order and memory of such models. However, these methods are quite complex methods, and they do not give an extraction of harmonic components *f*_0_, 2*f*_0_, 3*f*_0_,… and SUH components (*f*_0_/2)2, (3/2)*f*_0_, (5/2)*f*_0_,… since they model all spectral components (*f*_0_/2)2, *f*_0_, (3/2)*f*_0_, 2*f*_0_, (5/2)*f*_0_, 3*f*_0_,….

In order to reduce the complexity of such methods and to extract SUH components from all spectral components, we propose two new original approaches neither based on the Volterra filtering but based on the Hammerstein filtering.

This paper is organized as follows: after recalling standard Hammerstein model, the new methods are presented. To validate our methods, we propose realistic simulations of contrast agents signals. Then a quantitative and qualitative comparison is made between the two proposed methods with respect to standard Hammerstein model. Finally a discussion completed by a conclusion closed the paper.

## 2. Methods

**Figure 3:**
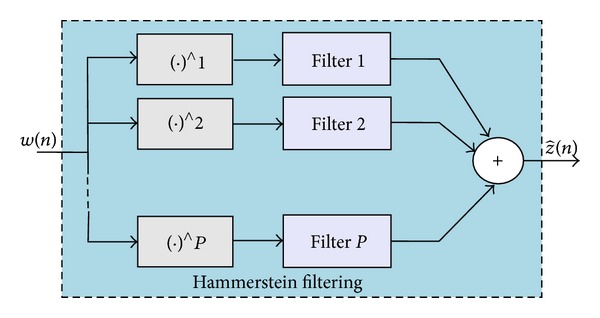
Block diagram of Hammerstein model.

Polynomial Hammerstein model is a special type of nonlinear filters in which a static nonlinear system is followed by a dynamic linear system [15]. From this model, the nonlinear system is approximated by a polynomial function, and the linear part is a finite impulse response (FIR) filter. The block diagram of Hammerstein model is shown in Figure 3. 

 As for the Volterra decomposition, the Hammerstein decomposition is able to model harmonic components, but it was unable to model sub- and ultraharmonic (SUH) components. Before explaining how it was possible to model SUH components, we recall the Hammerstein decomposition.

### 2.1. Hammerstein Decomposition

The Hammerstein modeled signal $\hat{z}(n)$ can be seen as the summation of *P* signals *z*_*p*_(*n*) coming from *P* parallel branches:

(1) $$\begin{array}{c} \hat{z}\left(n\right)=\sum_{p=1}^{p}z_{p}\left(n\right). \end{array}$$

In each branch, the signal *z*_*p*_(*n*) is the output of a linear filter *h*_*p*_(*n*) of input *w*_*p*_(*n*):

(2) $$\begin{array}{c} z_{p}\left(n\right)=\sum_{m=1}^{M}h_{p}\left(m\right)\cdot w^{p}\left(n-m\right)=w_{p}{\left(n\right)}^{T}h_{p} \end{array}$$

with **h**_*p*_ = [*h*_*p*_(1),…, *h*_*p*_(*M*)]^*T*^ and **w**_*p*_(*n*) = [*w* ^*p*^(*n* − 1),…, *w* ^*p*^(*n* − *M*)]^*T*^. Note that the input filter signals *w*_*p*_(*n*) = *w* ^*p*^(*n*) are obtained from a polynomial function. Finally, ∀*n* ∈ [*M* + 1, *M* + 2,…, *N*], where *N* is the length of the input signal *w*(*n*) and the vector signal of each branch *p* can be written by

(3) $$\begin{array}{c} Z_{p}=W_{p}^{T}h_{p} \end{array}$$

with **Z**_*p*_^*T*^ = [*z*_*p*_(*M* + 1), *z*_*p*_(*M* + 2),…, *z*_*p*_(*N*)], and **W**_*p*_ = [**w**_*p*_(*M*), **w**_*p*_(*M* − 1),…, **w**_*p*_(*N*)]. The output signal $\hat{z}(n)$ of the model could be written in a vector form $\hat{Z}$ as follows:

(4) $$\begin{array}{c} \hat{Z}=WH \end{array}$$

with **H** = [**h**_1_^*T*^, **h**_2_^*T*^,…, **h**_*P*_^*T*^]^*T*^ and **W** = [**W**_1_^*T*^, **W**_2_^*T*^,…, **W**_*P*_^*T*^]. To determine directly the filter parameters **H** from (4), we have to minimize the mean square error given by

(5) $$\begin{array}{c} \arg\underset{h_{p}}{\min}\left(𝔼\left[{\left(z-\hat{z}\right)}^{2}\right]\right), \end{array}$$

where *z* is the output signal of the non linear system and *𝔼* is the symbol of the mathematical expectation. The corresponding solution is

(6) $$\begin{array}{c} H={\left(W^{T}W\right)}^{-1}W^{T}Z, \end{array}$$

if (**W** ^*T*^ **W**) is invertible. Otherwise, regularization techniques can be used.

Consequently, identifying a nonlinear system of input *x*(*n*) and output *z*(*n*) with an Hammerstein model is equivalent to calculate the signal $\hat{z}(n)$ using (4) and (5).

### 2.2. Sub- and Ultraharmonics Modeling

**Figure 4:**
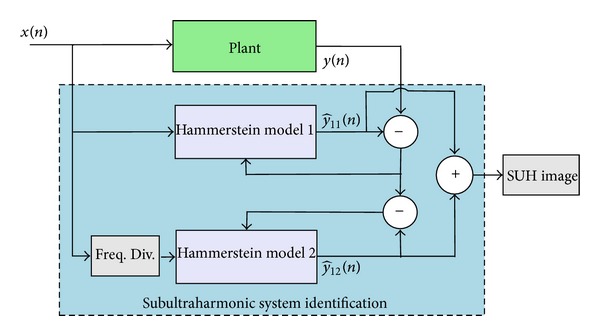
Block diagram of method  1, modeling by input frequency downshifting.

As previously mentioned, Hammerstein model is able to model harmonic components only. This is justified by the steady state theorem reported in [16] which stipulates that the output response of the model to a periodic input of frequency *f*_0_ is also a periodic signal of fundamental frequency *f*_0_. Suppose that the transmitted signal *x*(*n*) is periodic of frequency *f*_0_, and that the signal backscattered by the contrast agent has the following spectral components (*f*_0_/2)2, *f*_0_, (3/2)*f*_0_, 2*f*_0_, (5/2)*f*_0_, 3*f*_0_,…. Let ${\hat{y}}_{11}(n)$ be the output signal of the Hammerstein model of input *x*(*n*) (see Figure 4). According to the theorem reported in [16], the spectral content of the output signal ${\hat{y}}_{11}(n)$ is composed of the following harmonic components *f*_0_, 2*f*_0_, 3*f*_0_,… if and only if the input frequency is *f*_0_.

Thus, modeling SUH components of frequency *f*_0_/2, (3/2)*f*_0_, (5/2)*f*_0_,… using Hammerstein model is possible if and only if these components could be seen as integer multiples of the input frequency to the Hammerstein model. To do this, some modifications must be performed. Two types of modifications are proposed in this work, either at the input or at the output. Based on these two types of modifications, two methods for modeling and separating the sub- and ultraharmonic components using Hammerstein models are described in this section.

Each of the two proposed methods consists of two steps: one step for harmonic modeling and another step for SUH modeling. The first method is based on the modification of the input frequency, while the second one is based on the modification of the output frequency.

#### 2.2.1. Method  1: Modeling by Input Frequency Shifting

As previously mentioned, this method consists of two steps; each step uses one Hammerstein model as presented in Figure 4.

(1) Harmonic modeling: harmonic modeling is done by identifying the system of input *x*_11_(*n*) = *x*(*n*) and output *y*(*n*) with an Hammerstein model. The obtained signal ${\hat{y}}_{11}(n)$ has the harmonic components only. Referring to (4), the harmonic signal ${\hat{y}}_{11}(n)$ could be written as

(7) $$\begin{array}{c} {\hat{Y}}_{11}=X_{11}H_{11} \end{array}$$

with *X*_11_ and *H*_11_ defined as in Section 1.

(2) sub- and ultraharmonic modeling: the SUH information is found in the difference signal *y*_21_(*n*) between the output signal *y*(*n*) and the harmonic signal ${\hat{y}}_{11}(n)$:

(8) $$\begin{array}{c} y_{21}\left(n\right)=y\left(n\right)-{\hat{y}}_{11}\left(n\right). \end{array}$$

The spectral content of *y*_21_(*n*) is composed of *f*_0_/2, (3/2)*f*_0_, (5/2)*f*_0_,… by referring to the previous theorem; these components could be modeled using Hammerstein model if the input frequency is *f*_0_/2. Consequently, the initial input signal *x*(*n*) must be modified in such a way to bring up the subharmonic frequency *f*_0_/2. To do this, the spectrum of *x*(*n*) is downshifted by *f*_0_/2 to shift the frequency *f*_0_ toward the position of *f*_0_/2. The modified input *x*_12_(*n*) is calculated according to the following equation:

(9) x12(n)=ℜ((x(n)+jx~(n,))·e−2πj(f0/2)nTs)=x(n)cos⁡(2πf0nTsf02)+x~(n)sin(2πnTsf02),

where *ℜ* is the real part, $\tilde{x}(n)=ℋ(x(n))$ is the Hilbert transform of *x*(*n*), and *T*_*s*_ is the sampling frequency.

The modified input signal *x*_12_(*n*) could be written in the following vectorial form:

(10) $$\begin{array}{c} X_{12}=X_{C}+{\tilde{X}}_{C}, \end{array}$$

with

(11) XC=[x(1)cos⁡(2πf02Ts)x(2)cos⁡(2π2f02Ts)    ⋯x(N)cos⁡(2πNf02Ts)],X~C=[x(1)cos⁡(2πf02Ts)x(2)cos⁡(2π2f02Ts)    ⋯x(N)cos⁡(2πNf02Ts)].

Then, the SUH signal ${\hat{y}}_{12}$ is the output of a Hammerstein model that identifies the system of input *x*_12_(*n*) and output *y*_12_(*n*). In the same way, ${\hat{y}}_{12}(n)$ is calculated according to the following equation:

(12) $$\begin{array}{c} {\hat{Y}}_{12}=X_{12}H_{12}. \end{array}$$

Finally, the total modeled signal $\hat{y}(n)$ is the sum of the harmonic signal ${\hat{y}}_{11}(n)$ and the sub- and ultraharmonic signal ${\hat{y}}_{12}(n)$:

(13) $$\begin{array}{c} \hat{y}\left(n\right)={\hat{y}}_{11}\left(n\right)+{\hat{y}}_{12}\left(n\right). \end{array}$$

For this method, note that the maximal frequency that could be modeled is limited by the order *P* of the Hammerstein model. It is well known that the Hammerstein model of order *P* excited by a signal of frequency *f*_0_ can model harmonic components until the *P* ^th^ harmonic of frequency *Pf*_0_. In the case of ultrasound contrast agent and for some conditions explained later, the subharmonic component is (*f*_0_/2). In the second step of method  1, the *P* ^th^ harmonic is *P*(*f*_0_/2). For example, modeling *y*_21_(*n*) with an Hammerstein model of order *P* = 3 and input *x*_mod⁡_(*n*) of frequency (*f*_0_/2), calculated using (19), can model sub- and ultraharmonics until (3/2)*f*_0_. Therefore, to model all the sub- and ultraharmonic components presented in the signal *y*_21_(*n*), the order of the Hammerstein model must be adjust by increasing to *P* = 5.

#### 2.2.2. Method  2: Modeling by Output Frequency Shifting

**Figure 5:**
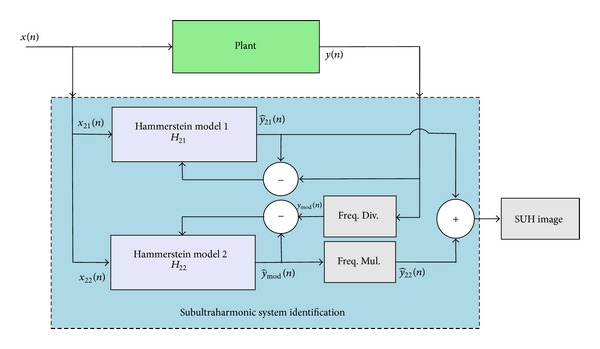
Block diagram of method  2: modeling by output frequency shifting.

This method also consists of two steps, on step dedicated for harmonic modeling and another step dedicated for SUH modeling. Each step uses one Hammerstein model as presented in Figure 5.

(1) Harmonic modeling: this step is the same as the first step of the method  1. The signal *y*(*n*) is modeled with a Hammerstein model of input *x*_21_(*n*) = *x*(*n*). The obtained signal is the harmonic signal ${\hat{y}}_{21}(n)$ calculated according to

(14) $$\begin{array}{c} {\hat{Y}}_{21}=X_{21}H_{21}. \end{array}$$

(2) Sub- and Ultraharmonic modeling: based on the same idea as reported in method  1, SUH components could be modeled when they are considered as integer multiples of the input frequency. In this method, we propose to keep the input signal *x*(*n*) and to change the output signal *y*(*n*) by upshifting its spectrum of *f*_0_/2. Now, the SUH components are shifted toward the harmonics position, and then they could be modeled with a Hammerstein model. The modified output signal *y*_mod⁡_(*n*) is calculated according to the following equation:

(15) ymod⁡(n)=ℜ((y(n)+jy~(n))·e2πj(f0/2)nTs)=y(n)cos⁡(2πnTsf02)−y~(n)sin(2πnTsf02).

In vector form,

(16) $$\begin{array}{c} Y_{\mathrm{mod}}=Y_{C}+{\tilde{Y}}_{C}, \end{array}$$

with

(17) YC=[y(1)cos⁡(2πf02Ts)y(2)cos⁡(2π2f02Ts)   ⋯y(N)cos⁡(2πNf02Ts)]Y~C=[−y~(1)sin(2πf02Ts)−y~(2)sin(2π2f02Ts)   ⋯−y~(N)sin(2πNf02Ts)].

Then the signal *y*_mod⁡_(*n*) is modeled with a Hammerstein model of input *x*(*n*). The obtained signal ${\hat{y}}_{22_{\mathrm{mod}}}(n)$ is calculated according to the following equation:

(18) $$\begin{array}{c} {\hat{Y}}_{\mathrm{mod}}=XH_{\mathrm{mod}}. \end{array}$$

has SUH components upshifted by *f*_0_/2. To recover the sub- and ultraharmonic signal ${\hat{y}}_{22}(n)$, the signal ${\hat{y}}_{22_{\mathrm{mod}}}(n)$ is downshifted of *f*_0_/2 according to the following equation:

(19) y^22(n)=ℜ((y^mod⁡(n)+jy^~mod⁡(n))·e−2πj(f0/2)nTs)=y^mod⁡(n)cos⁡(2πnTef02) +y^~mod⁡(n)sin(2πnTsf02).

The final signal $\hat{y}(n)$ is the sum of the harmonic signal ${\hat{y}}_{11}(n)$ and the sub- and ultraharmonic signal ${\hat{y}}_{22}(n)$:

(20) $$\begin{array}{c} \hat{y}\left(n\right)={\hat{y}}_{21}\left(n\right)+{\hat{y}}_{22}\left(n\right). \end{array}$$

## 3. Simulations and Results

To validate the two proposed methods and to quantify their performance in ultrasound medical imaging, realistic simulations are proposed. To achieve these simulations, the free simulation program Bubblesim developed by Hoff [17] was used to calculate the scattered echoes for a specified contrast agent and excitation pulse. A modified version of Rayleigh-Plesset equation was chosen. The model presented by Church [18] and then modified by Hoff [17] is based on the theoretical description of contrast agents as air-filled particles with surface layers of elastic solids. In order to simulate the mean behavior of a contrast agent cloud, we hypothesized that the response of a cloud of *N* contrast agents was *N* times the response of a single contrast agent with the mean properties.

The incident burst is a sinusoidal wave composed of 18 cycles of frequency *f*_0_ = 4.5 MHz and pressure of 0.6 MPa [13]. (The resonance frequency of the encapsulated contrast agent of 1.5 *μ*m is about 2.5 MHz. The emission frequency at 4.5 MHz is nearly the double of the resonance frequency.) Under the previous conditions of frequency and pressure, the oscillation of the contrast agent is nonlinear with sub- and ultraharmonic generation. The sampling frequency is *f*_*s*_ = 1/*T*_*s*_ = 60 MHz. The parameters of the contrast agent are given in the Table 1.

In this research work, the performances of the different methods are evaluated qualitatively and quantitatively.

### 3.1. Qualitative Evaluation

**Figure 6:**
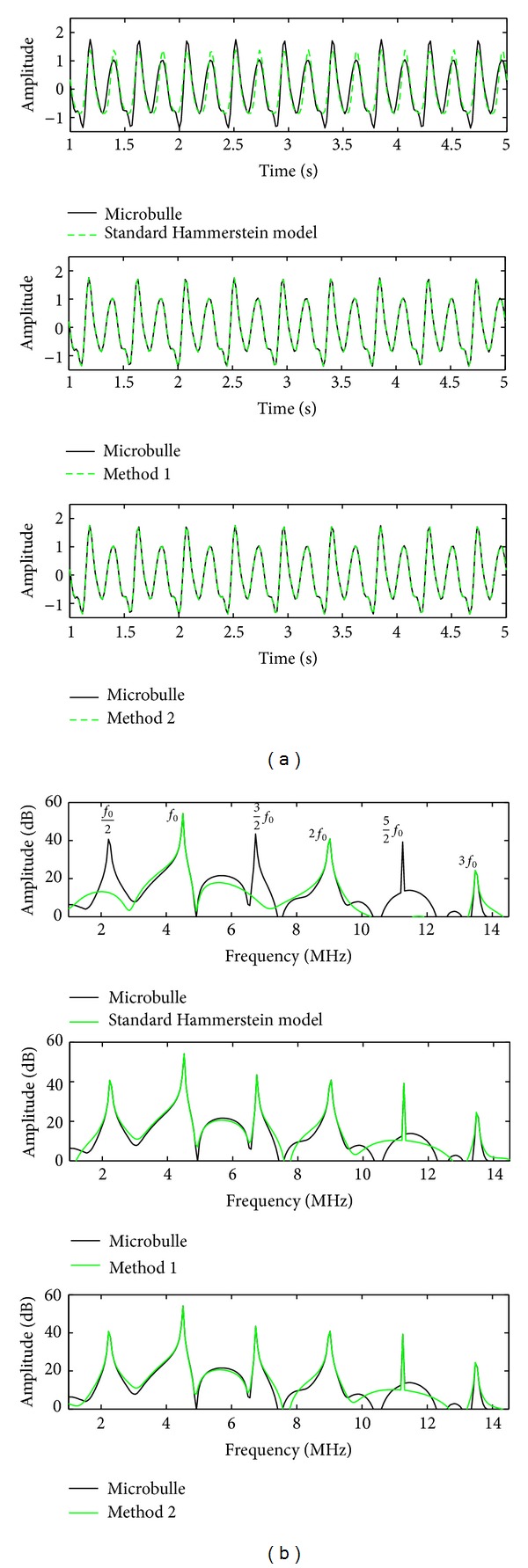
(a) Comparison between the signal backscattered by the contrast agent *y*(*n*) (in black) and its estimation $\hat{y}(n)$ (in green): the signal modeled with (top) the standard Hammerstein model, (middle) method  1, and (bottom) method  2. (b) Spectra of different signals presented in (a). Here SNR = *∞* dB.

Figure 6 represents a qualitative comparison in both time and frequency domains between the signal backscattered by the contrast agent *y*(*n*) and the signal obtained with the standard Hammerstein model, method  1, and method  2. Method  1 is applied with a Hammerstein model of order *P* = 3 and memory *M* = 30 for the first step and a Hammerstein model of order *P* = 5 and memory *M* = 30 for the second step. Method 2 is applied with a Hammerstein model of order *P* = 3 for the two steps and memory *M* = 30.

Figure 6(a) (top) shows that the modeled signal with the standard Hammerstein model does not describe correctly the signal backscattered by the contrast agent. Corresponding spectra in Figure 6(b) (top) show that the signal modeled with the standard Hammerstein model has the harmonic components only (*f*_0_, 2*f*_0_, and 3*f*_0_). Figure 6(b) (middle, bottom) shows that the signals modeled with methods  1 and 2 perfectly describe the contrast agent signal. Corresponding spectra on Figure 6(b) (middle, bottom) show that all the frequency components are modeled: harmonics at (*f*_0_, 2*f*_0_, 3*f*_0_), subharmonic *f*_0_/2, and ultraharmonics ((3/2)*f*_0_, (5/2)*f*_0_).

**Figure 7:**
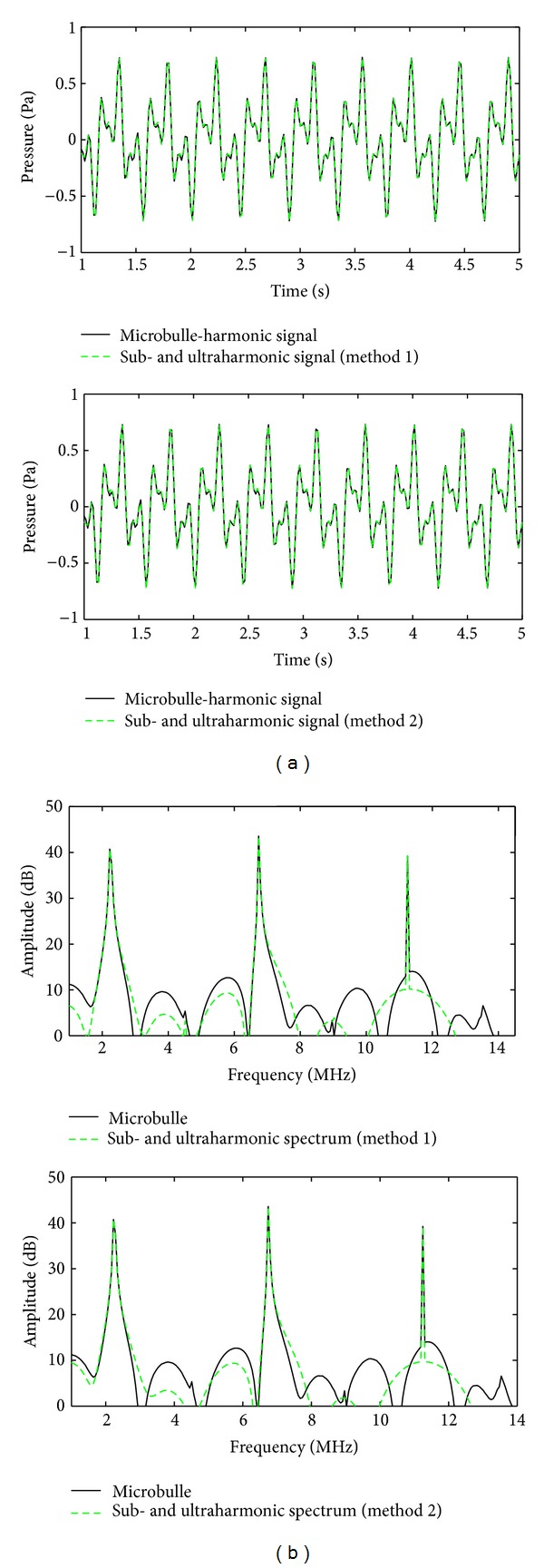
(a) Comparison between the backscattered difference signal between the backscattered signal by the contrast agent (black) and the SUH signal (green) modeled with (top) method  1 and (bottom) method  2. (b) Spectra of different signals presented in (a). Here SNR = *∞* dB.

Figure 7(a) (top) shows the different signal between *y*(*n*) and the harmonic signal (in black) and the sub- and ultraharmonic signal obtained with method  1 (top) and method  2 (bottom). We can see the good agreement between the signals. Spectra on Figure 7(b). (top) confirms that subharmonic *f*_0_/2, first ultra-harmonic (3/2)*f*_0_, and second ultraharmonic (5/2)*f*_0_ are well modeled and separated from other harmonic components. These results confirm the efficiency of the two proposed methods in modeling and separating the sub- and ultraharmonics present in contrast agent response.

#### 3.1.1. Quantitative Evaluation

**Figure 8:**
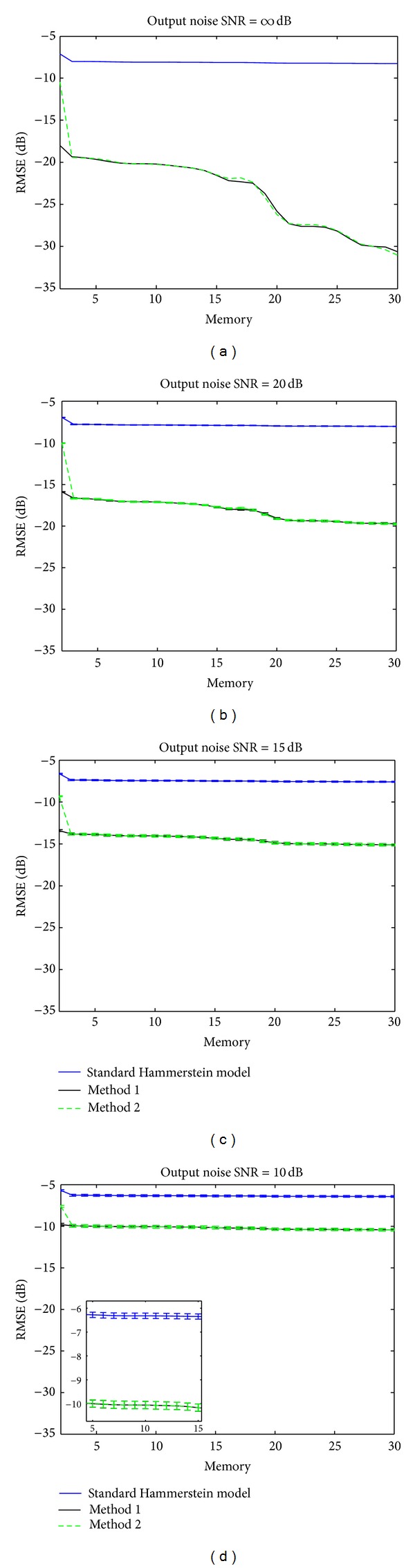
Variation of RMSE in dB between the backscattered signal by the contrast agent and that modeled with (blue) the standard Hammerstein model, (black) method  1, and (green) method  2 as a function of the memory of Hammerstein model in presence of output noise: SNR = *∞* dB, SNR = 20 dB, SNR = 15 dB, and SNR = 10 dB.

To quantify the performance of each method and to know which method provides the best performance, a quantitative study is necessary. The relative mean square error EQMR defined as

(21) $$\begin{array}{c} \text{RMSE}=\frac{E\left[{\left|\hat{y}\left(n\right)-y\left(n\right)\right|}^{2}\right]}{E\left[{\left|y\left(n\right)\right|}^{2}\right]} \end{array}$$

is evaluated for different noise levels at the system output. The noise level adjusted as the function of SNR (signal to noise ratio) is Gaussian and white. Ten realizations are made to evaluate the fluctuations of RMSE. RMSE for SNR = *∞*, 20,15, 10 dB is reported in Figure 8.

 These simulations show that the RMSE achieved with the two proposed methods  1 and 2 is always less than the RMSE achieved with the standard Hammerstein model for the different SNR values.

The gap between the standard model and the two methods  1 and  2 decreases when the SNR value increases. A gap ranging from 4 to 26 dB could be obtained depending on the SNR conditions. These results confirm that the standard Hammerstein model is not adapted for sub- and ultraharmonic modeling. A zoom on Figure 8(d) shows that the RMSE varies slightly around a mean value. This result shows that the two methods  1 and  2 are robust toward noise. Note that the curves of variation of RMSE obtained with the two methods have the same trend, indicating that the two methods tend toward the optimal solution.

Table 2 sums up the RMSE values obtained with the standard Hammerstein model, method  1, and method  2 when the SNR = *∞*.

## 4. Discussions and Conclusions

In this research work the problem of modeling sub and ultra-harmonics with Hammerstein model is presented. Usually, the standard Hammerstein model is able to model harmonics only, which are integer multiples of the input frequency. Sub and ultra-harmonics could not be modeled.

In this work, we propose for the first time two new methods that use Hammerstein filters that model sub and ultra-harmonics. The two methods are based on the same idea stipulating that modeling SUH with Hammerstein model is possible if the input signal or the output signal is modified In such a way that the SUH components become in the position of integer multiples of the input frequency.

Each method uses two Hammerstein models successively. The first one is dedicated to model harmonic components and the second one to model the SUH components.

The first method (method  1) applies a spectral downshifting of *f*_0_/2 on the input signal of the Hammerstein model. Now, SUH are seen as integer multiples of the input frequency, and therefore they can be modeled with Hammerstein model. In this step, the order of Hammerstein model is an important parameter that need to be adjusted to ensure the modeling of all SUH. For the second method (method  2), a spectral upshifting of *f*_0_/2 is applied on the output signal to move the SUH components toward the harmonic positions. Then, SUH components can be modeled with Hammerstein model excited with the input signal of frequency *f*_0_. Finally, a last spectral downshifting is performed to recover the SUH signal.

The two proposed methods are characterized by its simplicity. The originality of these methods is that they allow for the first time both the modeling of contrast agents signals and the separation of SUH components of the contrast agent response.

However, the two methods do not present the same advantages and disadvantages.

Method 1, which is based on the modification of the input signal, is less sensitive to the noise compared to method 2, which is based on the modification of the output signal. This is due to the fact that all the noise generated in the different parts of the non linear procedure are added to the output.

On the other hand, although method 1 has a more simple structure, it is slower than method 2. This is due to the fact that the second step of method 1 requires an order higher than method 2, the order of the first step being fixed. And as the computation time is related to the order of the model, higher the order, the slower the method.

The application of the proposed methods for modeling the contrast agents response shows their efficiency in modeling and in separating SUH components from other harmonic components. Gains of 25.8 dB and 22.8 dB in term of the RMSE are achieved with methods  1 and 2, respectively, compared to the standard Hammerstein model. The achieved error (RMSE) gain by the two methods is related to the sub and ultraharmonics energy initially presented in the output signal of the non linear system. The more important the energy of the sub- and ultraharmonics, the more important the gain. Although in this paper, the two methods works well for *f*_0_/2, (3/2)*f*_0_, (5/2)*f*_0_, these methods can be extended for other orders.

The two proposed methods find theirs applications in the field of sub and ultra-harmonic contrast imaging in order to produce high contrast images. This work opens a new research axis for new modeling techniques of SUH using Hammerstein model or any other non linear models.

## Figures and Tables

**Table 1:** The parameters of contrast agent.

Resting radius	*r*_0_ = 1.5 *μ*m
Shell thickness	*d*_Se_ = 1.5 nm
Shear modulus	*G*_*s*_ = 10 MPa
Shear viscosity	η = 1.49 Pa·s

**Table 2:** RMSE between the signal backscattered by the contrast agent and that modeled with the Hammerstein model, method 1, and method 2.

	Standard Hammerstein	Method 1	Method 2
RMSE (dB)	−8.3	−30.5	−31.1
